# Supplementary material for: Are carbon emissions trading and green financial instruments synergistic? -Comprehensive quantitative research based on content analysis
Source: PLoS One. 2024 Mar 7;19(3):e0298601. doi: 10.1371/journal.pone.0298601 (PMC10919601; doi:10.1371/journal.pone.0298601)
Supplement: S1 Data — (ZIP) [file pone.0298601.s001.zip › Data/地级市Z_F工作报告.zip!/╒π╜¡/╔▄╨╦╩╨/└┤╫╘╣1⁄2╓┌║┼í╛┬φ┐╦╩2╛▌═°í┐_2017.docx]

各位代表：

　　现在，我代表市人民政府向大会作工作报告，请予审议。并请市政协委员和其他列席人员提出意见。

过去五年回顾及2016年主要工作

2012年以来，本届政府在中共绍兴市委的坚强领导下，认真贯彻中央和省委省政府各项决策部署，坚持以生态文明建设为统领，以科学发展、执政为民为己任，积极进取、奋发有为，较好地完成了市七届人大一次会议确定的目标任务，各项事业取得新进展。

　　五年来，我们全力保增长促转型，经济发展稳中有进。2016年，全市实现生产总值4710亿元，年均增长7.7%。一般公共预算收入达到390亿元，年均增长10.2%。完成固定资产投资2882亿元，年均增长15.1%。实现社会消费品零售总额1783亿元，年均增长12.1%。自营出口总额达到1686亿元，保持全国12‰的份额。规模以上工业总产值突破1万亿元，高新技术企业由289家增加到754家，研究与试验发展经费支出占生产总值比重由1.9%提高到2.3%，发明专利授权量年均增长24%，新兴产业增加值占规上工业比重提高到32.8%。建筑现代化、住宅产业化水平不断提升，成为全国“双试点”城市。粮食生产保持稳定，高效生态农业加快发展，新建现代农业园区98个、粮食生产功能区92.5万亩，农业现代化综合评价指数居全省前列。服务业增加值占生产总值比重年均提高1.3个百分点，旅游总收入年均增长15.2%，柯桥兜率天景区、上虞杭州湾海上花田等项目建成投运，成为全国旅游标准化示范城市。绍兴滨海新城初出形象，柯桥经济技术开发区、杭州湾上虞经济技术开发区升格为国家级开发区。

　　五年来，我们聚力抓统筹促融合，城乡建设协调推进。市辖区行政区划调整顺利实施，城乡基础设施进一步完善，杭绍城际铁路、杭绍台铁路、金甬铁路、杭绍台高速公路等重大工程加快推进，嘉绍大桥、杭绍甬和杭长客运专线、灵绍特高压、高铁绍兴站、上虞中心大道、诸暨市区三环线等重大工程建成运行。镜湖新区初显框架，文化中心、科技中心、奥体中心投入使用。古城保护利用切实加强，功能疏解有序推进，浙东古运河绍兴段成功申遗。全市域协同扎实推进，诸暨中心城市格局加快拓展，嵊新区域协同发展成效明显。小城市、中心镇分类培育机制逐步健全，黄酒、袜艺、领尚等33个特色小镇建设全面推开。美丽乡村“四级联创”扎实开展，乡村旅游、民宿、农家乐等富农产业蓬勃发展。农村生活污水治理三年行动计划按期完成，农村饮水安全条件改善受益人数达到54.3万，生活垃圾集中处理行政村覆盖率达到95%。

　　五年来，我们大力强整治促生态，环境质量日益优化。环境主体功能区规划编制实施，国家森林城市成功创建，诸暨、新昌成为国家级生态县。排污许可“一证式”管理、印染废水分质提标集中预处理、生态环境大数据建设等全国试点深入开展，生态环境损害赔偿省级改革试点推进实施，汤浦水库和曹娥江流域生态补偿制度逐步完善。“五水共治”广泛深入开展，四级“河长制”体系全面建立，累计整治河道1253公里，连续四年成为全省交接断面水质考核优秀市，成功夺得“大禹鼎”。空气污染治理持续发力，淘汰企业落后产能3858家，完成印染企业煤改气178家、热电企业脱硫脱硝改造24家，淘汰黄标车4.8万辆，空气质量优良率达到79%。“三改一拆”强势推进，累计改造旧住宅区、旧厂区和城中村6663万平方米，拆违5503万平方米，拆后利用率达到70%。“两路两侧”“四边三化”专项整治成效明显。工业污泥、建筑泥浆、工程渣土实现“五统一”管理和市场化运作、资源化利用。节能减排任务顺利完成，单位GDP能耗累计下降21.5%。公安环保联合执法力度加大，查处违法案件3933件。

　　五年来，我们着力抓改革促开放，发展活力不断增强。积极开展各项深化改革试点，新昌县域科技体制改革、柯桥企业投资项目高效审批、上虞防范企业融资风险体制机制创新等在全国或全省推广。“放管服”改革深入推进，“四张清单一张网”编制完成，削减和调整市级行政审批事项508项。要素配置市场化、企业综合评价分类管理、商事登记制度和“营改增”税制改革等顺利实施。企业上市持续推进，新增上市公司13家、新三板挂牌企业61家。农村集体资产股份制改革全面完成，市县乡三级农村产权交易平台实现全覆盖。户籍新政全面实施，新型居住证制度开始推行。主动融入国家“一带一路”倡议，首届国际友城大会成功举办，对外交流、投资合作和越商回归工作扎实推进，五年累计利用外资41.7亿美元，完成境外投资38.4亿美元，越商回归投资超过1000亿元。援疆、援藏、援青和山海协作任务全面完成。

　　五年来，我们努力重民生促保障，人民生活持续改善。民生投入力度持续加大，全市民生支出占公共财政支出比例达到74.9%。创业就业活力不断增强，城镇登记失业率始终控制在3%以内。居民创收增收计划有效推进，城镇和农村常住居民人均可支配收入分别达到50305元和27744元，年均增长9.1%和10%。经济薄弱村和低收入农户奔小康结对帮扶活动不断深化，家庭人均年收入4600元以下农户全部实现脱贫。住房保障体系更加健全，累计新建保障性住房10.2万套。义务教育标准化学校创建率全省第二，课程改革不断深化，新增高校4所，柯桥、上虞创建成为省教育基本现代化区。基层卫生综合改革、公立医院改革、卫生信息化等全国试点扎实推进，分级诊疗有序开展，建成“双下沉、两提升”医联体37家。城乡居民大病保险、残疾人意外伤害保险制度全面实施，全民参保登记率达到100%，国家卫生城市通过复查。“两孩”政策稳步实施，人均期望寿命提高到80.6岁。智慧居家养老服务标准化试点扎实开展，养老机构床位数增加到每百人4张。红十字、残疾人事业健康发展，公益慈善指数排名居全省首位。文化强市建设加快推进，创建成为全国文明城市。文化惠民持续深化，建成农村文化礼堂519个。群众体育和竞技体育全面发展，全民健身广泛开展，第十五届省运会成功承办。

　　五年来，我们致力严治理促和谐，社会保持平安稳定。“枫桥经验”不断传承发展，立体化治安防控体系更加健全，信息化实战警务成效明显，G20杭州峰会和世界互联网大会等维稳安保任务圆满完成。“六五”普法深入开展，法治政府建设纲要制定实施。统一政务咨询投诉平台建立运行，全市信访总量和刑事案件总数持续下降，群众安全感满意率达到96.2%。城市管理综合执法、食品药品监管体制不断完善。安全生产责任制有效落实，事故起数、死亡人数连年下降。气象灾害防御体系逐步健全，H7N9流感等重大疫病有效防控，应急管理水平扎实提升。中央“八项规定”精神全面落实，廉洁政府建设不断加强。公车改革有序推进，公务考察、公务商务接待、办公用房和编外人员管理规范执行，“三公”经费支出、因公出国（境）人次分别下降58.9%和34%。国防动员、民兵、双拥、人防、国家安全等工作取得新成绩，民族宗教、对台事务、档案、史志工作以及妇女、儿童等事业得到新加强。

　　刚刚过去的2016年，是本届政府的收官之年。面对艰巨的改革发展稳定任务，我们始终保持定力，坚定不移打好转型升级系列组合拳，实现了“十三五”发展的良好开局。一是稳增长。千方百计扩大有效投资，着力抓好“三去一降一补”，积极培育信息、教育、旅游等新消费，努力帮扶企业化解生产经营困难，全市生产总值增长5.5%，一般公共预算收入增长7.3%，规上工业增加值增长4.7%，第三产业增加值增长6.9%，固定资产投资增长11.6%，自营出口实现正增长。二是调结构。围绕“绿色高端、世界领先”目标，切实强化标准管控，停产整治印染企业107家、化工企业102家，整治提升“低小散”企业2671家，淘汰企业落后产能330家，处置僵尸企业28家。大力发展新兴产业，高新技术和装备制造业增加值占规上工业比重分别达到28.7%和29.9%。三是优环境。推动制定《绍兴市水资源保护条例》和《绍兴市大气污染防治条例》，深入实施“811”美丽绍兴建设行动计划。持续打好“五水共治”攻坚战，完成清淤1951万立方米，消灭省控以上五类水断面24个。开展燃煤烟气、工业废气、汽车尾气治理等专项行动，淘汰老旧机动车1.3万辆，空气质量改善幅度全省第一。全面打响治危拆违攻坚战，完成“三改”3229万平方米，拆违1973万平方米、完成率居全省首位。实施“深度清洁”城市保洁，启动城乡生活垃圾分类试点。四是促融合。调整完善越城区、高新区管理体制，逐步理顺市与区事权。实施基础设施相连相通计划，推进群贤路东延、104国道柯桥段高架改造等项目，建成孙曹公路、二环北路拓宽等工程，城市轨道交通一期建设规划获批。完成三区公交融合，完善干线公交、快速公交体系。搭建统一的智慧城管、智慧交管信息共享平台，清理规范主干道名称，制订旅游交通标识标牌一体化规划。建立三区统一的基本医疗保险制度，健全普通高中、中职教育融通招生机制。五是惠民生。市政府承诺的十方面民生实事如期完成。推广“就业通”线上服务平台，帮助城镇失业人员再就业4.2万人，培养高技能人才2.1万人。提高企事业单位退休人员养老金标准，完成机关事业单位养老保险制度改革，新增市民卡服务网点307个。统一市区城乡低保标准，提高全市医疗救助水平，建立残疾人生活、护理补贴制度。持续优化公共服务，新建社区居家养老服务照料中心826个，完成幼儿园建设50所，全科医生签约服务对象达到109万人。提高城乡交通品质，协同推进治堵工作，新建公交站点135个、农村公路候车亭379个，新增公交营运里程273公里，铁路始发列车实现零突破。新建“菜篮子”基地1040亩、改造提升1620亩，农产品抽检合格率达到99.5%。

　　各位代表，过去五年取得的成绩来之不易，这既得益于省委省政府和市委的正确领导，也是全市上下勠力同心、砥砺奋进的结果。在此，我谨代表市人民政府，向各位代表、委员，向各民主党派、工商联、人民团体和社会各界人士，向全市人民和所有关心支持绍兴发展的海内外朋友们，表示衷心的感谢！

　　与此同时，我们也清醒地看到绍兴经济社会发展中还存在不少困难和问题，主要是：经济平稳较快增长面临较大压力，2016年全市生产总值、外贸出口、居民收入增幅没有完成预期目标，产业层次不高，科技实力不强，经济发展质量有待提高；县域经济在新常态下遭遇要素配置、人才引进等瓶颈，统筹全市域资源要素力度不够，人文、生态、旅游资源优势转化为发展优势还要做更多工作；城乡协调发展的体制机制需要完善，市区集聚辐射功能亟需增强；一些改革举措落地见效不快，市场主体多元化程度偏低，外生性增长动力不足；生态环境还比较脆弱，生态治理任重道远；公共服务有待完善，公共安全、社会稳定还存在隐患；一些干部担当精神不够，能力建设还有薄弱环节，腐败问题仍有发生，等等。在今后的工作中，我们一定要高度重视这些问题，并采取有力措施切实加以解决。

今后五年的目标任务

　　今后五年，是充满机遇和挑战的五年。全市上下要清醒认识形势，强化忧患意识，深刻把握绍兴当前所处的历史方位，按照“秉持浙江精神，干在实处、走在前列、勇立潮头”的要求，努力推动绍兴在新一轮发展中取得更大作为。

　　新一届政府工作的指导思想是：高举中国特色社会主义伟大旗帜，以邓小平理论、“三个代表”重要思想、科学发展观为指导，认真学习贯彻习近平总书记系列重要讲话精神，深入践行新发展理念，以“八八战略”为总纲，坚持“拆治归”，持续深入打好转型升级系列组合拳，以生态文明建设为统领，强化创新驱动，深化改革开放，注重城乡统筹，聚焦民生改善，着力提升绍兴产业品质、城市品质、环境品质、文化品质、生活品质，全面加强政府自身改革和建设，勇立潮头、勇当标杆，为高水平全面建成小康社会而不懈奋斗。

　　今后五年的主要目标是：在高水平全面建成小康社会的基础上，确保全省第二方阵“排头兵”地位，奋力向第一方阵全面进发。全力打造经济转型升级的标杆、城乡统筹协调的标杆、生态环境优美的标杆、文化创新引领的标杆、社会和谐稳定的标杆和干部作风过硬的标杆。

　　今后五年按照市第八次党代会决策部署，重点做好以下六方面工作：

　　一是强化创新驱动，着力提升绍兴产业品质。大力振兴实体经济，以创新引领实体经济提质增效升级，推动经济综合实力迈上新台阶，到2021年，全市生产总值超过7000亿元、人均生产总值超过15万元、一般公共预算收入超过550亿元。深入实施纺织、化工、金属加工、黄酒、珍珠等传统产业改造提升省级试点，推广装配式建筑和住宅全装修。重点发展电子信息、高端装备、现代医药、新材料等新兴产业，积极发展军民融合产业，高新技术、新兴产业增加值占规上工业比重分别达到45%和40%以上。全面提高创新能力，加快建设绍兴科创大走廊，打造人才生态最优市，研究与试验发展经费支出占生产总值比重达到3%。大力促进服务业兴市，重点发展文化旅游、生命健康等现代服务业，积极发展工业设计、现代金融等生产性服务业，培育发展数字经济、智慧经济、分享经济等新业态新模式。推进农业供给侧结构性改革，大力发展品质农业，促进农业增效农民增收。

　　二是强化市域统筹，着力提升绍兴城市品质。加快推进全市域协同发展和市区融合发展，优化市域空间、产业、基础设施和公共服务布局，构建“一主两副多点”城镇空间体系，全市城镇化率达到75%左右。加快主城区融合发展，构建以镜湖新区为中心的大市区格局，统筹建设金融、教育、医疗、商贸设施，积极发展都市经济。高品质推进绍兴古城保护利用，有效疏解古城功能，打造独具江南水乡魅力和人文气息的城市风貌。推动诸暨组群“北承南接”，提升产业集聚和城市发展水平。深化嵊新组群协同协作，促进区域产业协同、基础设施共建、公共服务共享。完善基础设施，加快建设市区高速环线，建成杭绍台铁路、金甬铁路、杭绍甬高速、杭绍台高速及西线等工程。推进城乡交通一体化，实现“镇镇二级路、村村通公交”。加强城市品质营造和有序管理，加快“智慧绍兴”建设，基本实现全市无违建。优化村庄设计，全域全景建设美丽乡村。

　　三是强化生态治理，着力提升绍兴环境品质。坚定不移推进生态文明建设，创建国家生态文明建设示范市，绝不把脏乱差、污泥浊水、违法建筑带入全面小康。联动推进治水治气治土，全市所有水体达到三类水以上，空气质量优良率稳定在90%以上，除大型热电企业外实现全市域基本无煤化。大力推进节能生产和新型清洁能源替代，确保万元GDP能耗下降20%。推进全域创建国家森林城市，加快建设以会稽山国家森林公园、东鉴湖水乡风貌示范区为重点的“十大公园”，以曹娥江绿道、浦阳江绿道为重点的“十大绿道”，推进以镜湖、瓜渚湖为代表的“十大湖泊”景观提升，积极建设“花园城市”，全市森林覆盖率稳定在56%以上，城市建成区绿地率达到41%以上。完善生态文明建设制度体系，打造环保执法最严城市。

　　四是强化传承创新，着力提升绍兴文化品质。充分发挥稽山、鉴水、名人、书法、黄酒、越剧、绍剧等历史文化资源优势，弘扬大禹精神，活化阳明文化资源，提升鲁迅文学奖影响力。加强文献资料、文物古迹、文学艺术的保护传承，打造区域特色文化高地，文化及相关特色产业增加值年均增长15%以上，文化发展指数达到120，成为具有国际影响力的历史文化名城。推进文化体制改革，加快文化小康建设，实施基本公共文化服务标准化工程，建设一批具有地方特色的博物馆、艺术馆、美术馆，提高文化惠民水平。坚持社会主义核心价值观，树立崇德向善的良好风尚，提高文明城市建设水平。

　　五是强化共建共享，着力提升绍兴生活品质。积极创新社会治理，平安绍兴、法治绍兴建设水平走在全省前列，群众安全感满意率稳定在96%以上。坚持教育优先发展，普及学前教育，提高义务教育质量，推动普通高中多样化发展，深化职业教育产教融合，加强高等教育内涵建设，努力打造优质教育之城。推进“健康绍兴”建设，大力发展体育事业，完善养老服务体系，城乡医疗服务水平达到全省前列。实施更加积极的就业政策，创造更多的就业岗位，以创新促进创业，以创业带动就业。完善收入分配制度和劳动报酬增长机制，城镇和农村常住居民人均可支配收入分别达到7.5万元和4.5万元左右。建立健全公平、可持续的社会保障制度，基本养老保险、医疗保险实现人群全覆盖。健全社会救助体系，大力发展红十字和慈善事业。打造“枫桥经验”升级版，加快建设以枫桥镇为代表的平安浙江特色小镇。落实安全生产责任制，加强应急能力体系建设，提高防灾减灾救灾综合能力。

　　六是强化改革开放，着力提升发展活力动力。坚持市场化改革方向，着力健全市场在资源配置中起决定性作用和更好发挥政府作用的制度体系。积极支持民营企业创业创新，落实中小企业优惠政策，放活搞好小微企业。深化国企国资改革，推进国企整合重组，创新国资监管和营运体系。深化行政审批制度改革，推进“最多跑一次”改革。加快农村产权制度改革，全面深化户籍制度改革，促进有能力在城镇稳定就业和生活的常住人口有序实现市民化。推进社会领域改革发展，放开公共服务市场，引导社会资本进入公共服务领域。深化商事制度改革和市场监管体系改革，构建法治化、国际化、便利化的营商环境。全面融入“一带一路”、长江经济带战略，主动加强与周边都市区的融合对接，积极参与“义甬舟”开放大通道建设。推动外贸优进优出，促进外贸提质增效。大力培育本土民营跨国公司，支持企业瞄准先进技术和知名品牌开展跨国并购。

2017年主要工作

2017年是实施“十三五”规划的重要一年，是推进供给侧结构性改革的深化之年，也是新一届政府的开局之年，做好全年工作意义重大。建议全市主要发展预期目标为：生产总值增长7%以上，一般公共预算收入增长7.5%以上，固定资产投资增长10%以上，城镇和农村常住居民人均可支配收入分别增长7%、7.5%以上，城乡居民收入比控制在1.8以内，完成省下达的节能减排降碳任务。市政府将按照市委的总体部署，坚决打好“八大战役”，着力补齐“八块短板”，重点推进以下几方面工作：

　　（一）聚焦转型升级，振兴实体经济。坚持工业立市，持续深入打好经济转型升级系列组合拳，推进产业跃升发展，全年规上工业增加值增长7%以上。

　　改造提升传统产业。围绕纺织、化工、金属加工、黄酒、珍珠等重点产业，全面开展传统产业改造提升省级试点，着力拉长产业链、补强创新链、提升价值链，确保传统产业稳定健康发展。打好印染、化工落后产能歼灭战，按照“绿色高端、世界领先”目标，强化标准管控，完成182家印染企业、190家化工企业搬迁集聚和整治提升，全面推进452个工业园区整合治理，抓好柯桥印染产业省级转型升级示范区试点。实施黄酒产业原产地保护规划，精心举办黄酒节等产业展会活动，加快发展越窑青瓷、茶叶等历史经典产业。加强建筑产业集聚培育，建筑业增加值增长7%，新建装配式建筑比例达到15%。开展产业结构调整负面清单管理，淘汰企业落后产能300家以上。

　　大力发展新兴产业。加快培育电子信息、高端装备、现代医药、新材料等新兴产业，新兴产业增加值占规上工业比重达到35%以上。全面落实中国制造2025绍兴实施方案，推进工业化、信息化深度融合，推广移动互联网、云计算、大数据、物联网等创新应用。实施现代服务业行动计划，积极发展楼宇经济、总部经济等都市经济，加快发展工业设计、科技服务、现代金融、电商物流等生产性服务业，服务业增加值增长8%以上。实施文化旅游重点产业项目60个以上，旅游收入增长15%以上，文化旅游产业增加值达到570亿元。加快航空、e游等特色小镇建设，完成投资300亿元以上。争创首批省级军民融合创新示范区。

　　全力支持实体经济发展。积极营造重商、惠商、亲商氛围，加强政银企有效对接，着力缓解企业融资、技术、用工等难题，切实提振企业发展信心。深化企业减负降本行动，组建政策性融资担保公司，拓展涉企综合服务平台功能，努力把更多公共资源投入到实体经济发展中去。加快培育和壮大市场主体，力争新增市场主体6.8万家，培育“隐形冠军”企业200家。加大企业帮扶解困力度，坚持和深化企业服务员制度。完善风险企业、僵尸企业市场化退出机制，化解处置不良贷款230亿元以上。

　　（二）注重创新引领，增强发展动能。抢抓新一轮科技和产业变革战略机遇，促进创业创新，蓄积发展动能。

　　强化科技创新驱动。大力创建国家创新型城市、国家知识产权示范城市，加快建设新昌全面创新改革实验区，编制实施绍兴科创大走廊规划，推进“****”产业园、浙大网新上虞科技园、国科小镇等重点项目建设，确保研究与试验发展经费支出占生产总值比重达到2.5%。深化科技资金管理体制改革，构建科技创新基金、成果转化基金、金融风险池等多元投入体系。深入推进高新技术企业培育省级试点，新认定高新技术企业180家、省科技型中小企业680家。大力推动大众创业、万众创新，新建众创空间10家、省级以上研发机构30家，建成运营绍兴科技大市场。

　　打造区域人才高地。全面落实“人才新政”，搭建“一站式”人才服务平台，新建外国专家工作站8家、省级院士专家工作站2家，引进各类人才5.5万名，其中新引进海外高层次人才100名以上，引进国家和省“****”人才30名以上。弘扬绍兴企业家精神，实施民营企业家培训计划，造就一支勇立潮头、开拓进取的越商队伍。推进高技能人才振兴工程，培养一批具有工匠精神的高技能人才。

扩大各类有效投资。大力推进696个重大项目建设，当年项目开工率达到90%以上，完成年固定资产投资3200亿元，其中工业投资1400亿元以上，服务业投资增长15%。开工建设康龙化成医药中间体、苏泊尔生活电器、三花汽车零部件、东方山水二期等重点产业项目，建成投用卧龙电机、万丰摩轮、梅轮电梯等项目。实施基础设施投资“四大网络”工程，打好综合交通大会战，开工建设轨道交通1号线、高铁绍兴站枢纽、金甬铁路绍兴段等重大工程，加快推进杭绍台高速公路、杭绍城际铁路等项目建设，建成投用绍诸高速延伸线、杭金衢高速拓宽等项目。积极谋划跨钱塘江铁路桥等重大项目，提升杭甬运河、曹娥江航道通江达海功能。

　　（三）强化全域统筹，加快协同发展。坚持全市域协同大市区融合，着力推进新型城镇化，构建城乡均衡发展格局。

　　促进全域协同发展。编制实施新一轮城市总体规划（2016—2030），实现市域统筹、“多规合一”。推进诸暨“北承南接”战略，加快诸暨临杭产业园、诸义产业园建设。深化嵊新区域协同发展省级试点，谋划嵊新区域通道经济发展，完成嵊新污水处理厂扩建、上虞—嵊州—新昌天然气工程建设。优化中心镇布局，实施新一轮小城市培育创建工程。完善城乡交通网络，加快527国道嵊新段、市区与店口快速路等项目建设，实现全市公交“一卡通”。

　　加快三区融合步伐。围绕同城、一体目标，全面落实改革、规划、政策、项目等方面44项年度清单任务，加大三区连接区域开发建设力度。加快推进城市道路三年建设计划，构建城市快速路网体系。统筹谋划轨道交通沿线功能布局和设施配套，开工建设风情旅游新干线，加快推进群贤路东延、绍三线北延、绍兴汽车南站等工程建设。编制实施城市有机更新规划，优化城市空间开发利用，实现城市功能强化完善、城市形象美化提升。

　　推进新城开发和古城保护利用。编制实施镜湖新区控制性规划，加强城市设计引领，完善开发建设体制，把镜湖新区打造成为全市首位度的城市核心和地标性新城区。完善镜湖新区优质基础教育、医疗、商贸、交通等配套设施，加快接壤区域的连片拆改整治，做好“水”“绿”“景”文章，促进新城功能完善、人气集聚、商气营造。积极推进古城保护利用，加快古城功能疏解和风貌修复，实施特色街区复兴计划，推进书圣故里、西小路、越子城等历史街区保护工程建设，精心保护古城内亭、台、楼、阁、桥等历史建筑。推进东鉴湖及沿线保护整治，讲好绍兴故事，促进古城活化利用。

　　（四）夯实三农基础，建设美丽乡村。把“三农”工作放在更加突出位置，加快推进农业供给侧结构性改革，提升农业品质、农村环境和农民生活水平。

　　做强现代农业产业。稳定粮食生产，新建粮食生产功能区8.1万亩。深化农业现代园区建设，积极发展品质农业和生态循环农业，打造一批主导农业产业集聚区和特色农业龙头企业。加快畜牧业转型升级，新建省级美丽生态牧场34家。加快培育新型经营主体和服务主体，加强农民专业合作社规范化建设。推广农业科技创新，积极发展“互联网+农业”，加快打造农业全产业链。实施诸暨全国农村一二三产业融合发展试点，推进农业与制造业、服务业共生互融。

　　打造美丽乡村升级版。修编全市村庄布局规划，开展村庄设计。深化农村环境整治，推进美丽乡村、美丽田园建设，新建美丽乡村景观带10条，创建美丽乡村公路乡镇16个。加强对古镇、古村和农村历史建筑的保护，实施空心村、高山零星村、地质灾害隐患村撤并搬迁。完善农村生活污水处理设施运行管理，实施94个中心村电网升级，启动“村村通公交”三年行动，完成300个行政村公交通行提升改造。

　　拓展农民增收渠道。大力发展乡村旅游、家庭农场、“农家乐”、民宿经济和农村电商，创建A级景区村200个、3A级景区村20个。积极推进农村产权要素交易和融资，努力增加农民财产性收入，确保低收入农户人均可支配收入增长10%以上。增强经济薄弱村自我发展能力，消除村级集体收入10万元以下经济薄弱村。

　　（五）推动改革落地，增创发展优势。坚持问题导向、需求导向，全面深化重点领域和关键环节改革，提升资源配置效率与政务服务效能。

　　加快推进“最多跑一次”改革。以“最多跑一次”倒逼政府自身改革，市县镇同步实施、协同推进，为企业和群众提供一站式、跑一次、零距离的政府服务。实施“一窗受理、集成服务”改革，健全“一门办理、并联审批”机制，深化“市县同权、多级联动”服务模式，加强乡镇便民服务平台建设。加快政府核准投资项目目录“瘦身”，推进区域环评、能评试点改革，健全“订单式”企业投资项目高效审批服务机制，开展容缺受理审批服务，打造“中介超市”全市一张网。推广“互联网+政务服务”，基本实现便民服务事项网上办理全覆盖。

深化要素配置市场化改革。完善企业综合评价分类管理制度，健全资源要素差别化配置机制。做大做强上市企业绍兴板块，深化上市公司引领产业发展示范区试点，确保全市新增上市企业7家、新三板挂牌企业23家，新增社会融资1200亿元、直接融资600亿元。积极推进低效用地再开发，盘活存量用地7700亩，消化批而未供土地7800亩。加快国企混合所有制改革，组建多元产业基金和投资基金。

　　确保各项综合改革落地。完善产权保护制度，依法平等保护各类产权，增强各类经济主体创业创新动力。健全综合执法体系，推动城管考评向县（市）、镇（街）、社区延伸。深化基层治理体系改革，全面完成乡镇综治工作、市场监管、综合执法、便民服务等“四个平台”建设任务。深化户籍制度改革，推行流动人口IC卡居住证管理。加快土地“三权分置”改革，推进农民宅基地（农房）置换改革，推广嵊州农村承包土地经营权抵押贷款试点。

　　（六）扩大对外开放，提升发展活力。牢固树立“市外就是外”的大开放理念，提高对外开放质量和发展的内外联动性。

　　促进内外贸易转型发展。深化外贸优进优出战略，鼓励先进设备和关键技术进口，提高机电、高新技术和文化产品出口比重，全年自营出口增长4%。完善外贸便利化举措，全面实施“单一窗口”和通关一体化，加强外贸特殊功能区建设，大力发展新型加工贸易。制订“放心消费在绍兴”计划，推进专业市场、商贸综合体整合提升，确保社会消费品零售总额增长10%。

　　打好精准招商战。强力推进招商引资，深化专业招商、精准招商，完成产业项目招商500亿元以上，实际利用外资10亿美元以上。紧盯央企、军企、民企，瞄准上市企业和创新成长型企业，招引大项目好项目，争取50亿元以上产业项目有重大突破。充分发挥越商资源优势，强化以商引商、以商引智，推动越商产业、总部、资本、技术全方位多领域回归，加快新昌浙商跨国并购回归项目示范园建设，越商回归到位资金300亿元以上。推进开发区（园区）、产业集聚区等功能区整合提升，提高承载大产业、大项目能力。

　　深化区域经济合作。积极参与浙江自贸试验区、“义甬舟”“义新欧”开放大通道建设，深化与国际友城的经贸合作。主动接轨上海，积极融入杭州、宁波都市区，加强与温州、金华以及浙东合作区城市的对接合作。鼓励企业“走出去”并购优质资产、创建生产基地、构建营销网络，力争实现并购金额100亿元。继续做好对口支援、山海协作等工作。

　　（七）深化环境治理，建设生态绍兴。全面实施“811”美丽绍兴建设行动，联动推进治水治气治脏治乱，争创国家生态文明建设示范市。

　　深入实施“三改一拆”。打好城中村围剿战，全面推进106个城中村改造项目，完成拆改1000万平方米、改造旧住宅1000万平方米，拆违1000万平方米、拆后利用率达到80%，完成全市D级危房和涉及公共安全的 C级危旧房治理。建立健全覆盖所有镇街的长效防控机制，实现新增违建“零增长”。打好整治市容市貌和小城镇环境阵地战，确保全市30%的小城镇通过达标考核，各小城镇整治项目总体形象进度达到30%以上。深化“两路两侧”“四边三化”等专项行动，全面推进“深度清洁”，完成26个美丽门户创建，启动7个入城口改造。实施国土绿化“十大”工程，造林更新2万亩。打好垃圾分类处理持久战，确保市区、各县（市）城区生活垃圾分类总覆盖率分别达到70%和50%以上，农村生活垃圾分类行政村覆盖率达到60%以上，建成投运循环生态产业园一期项目。

　　纵深推进“五水共治”。打好劣五类水和五类水剿灭战，巩固深化河长制，大力实施治水“八大行动”，完成清淤1000万立方米以上，实现128个地表水水质考核断面自动监测全覆盖，三类以上水质断面比例达到80%以上，功能区达标率达到86%以上，全面消除劣五类水体和五类水断面，确保再夺“大禹鼎”。扎实推进防洪排涝“双十”工程，加快建设上虞姚江上游西排、诸暨高湖蓄滞洪区等重大水利项目，实现钦寸水库顺利蓄水。编制海绵城市专项规划，就地消纳利用降水75%以上。

　　重拳整治大气污染。加强工业废气、机动车尾气、建筑扬尘等综合整治，全面淘汰每小时10蒸吨以下燃煤锅炉，完成所有每小时65蒸吨以下燃煤锅炉清洁排放改造和26家热电企业超低排放改造。完善空气质量预报预警制度，在全市所有镇街建立PM2.5自动监测站点，确保全年空气质量优良率稳定在80%以上，其中一级天数达到100天。

　　建设生态文明建设示范市。建立市县两级生态建设委员会，强化市级环境监测和环境执法职责，统筹全市生态文明示范市建设和环境保护工作。加强能源消费总量和用能强度“双控”，推广应用新能源汽车等绿色低碳产品，实现原煤消费总量下降5%，规上工业增加值能耗下降5%。坚持铁腕治污，落实重大决策、区域开发、项目建设等环保一票否决制，建立“生态报表”发布制度。完善污染物排放财政收费、单位GDP能耗奖惩等制度，推广用能权、用水权、排污权、碳排放权市场交易。优化土壤环境监测点位布局，推进农业园区土壤污染治理修复。

　　（八）切实改善民生，促进和谐稳定。坚持以人民为中心，不断优化公共服务供给，努力让广大市民拥有更强的安全感、获得感和幸福感。

　　完善社会保障体系。实施高校毕业生就业促进计划，制订公益性岗位管理办法，新增城镇就业10万人，实现城镇失业人员再就业3.5万人。加快推动社保政策融合，统一全市生育保险待遇。坚持医养结合，扶持发展民办养老产业，推进乡镇敬老院转型升级，新增机构养老床位2500张，启动红十字参与养老服务国家试点，完成国家智慧居家养老标准化试点。提高医保基金使用效率，推进医保付费方式改革。完善最低生活保障、临时救助、社会慈善“三位一体”托底帮扶体系，拓展困境儿童、留守儿童保障范畴。推进社会化优抚工作，继续创建全国双拥模范城市。

　　加快社会事业发展。推进教育优质均衡发展，实施学前教育新三年行动计划，建成投用绍兴一中新校区、新昌技师学院、浙江理工大学科学与艺术学院，加快绍兴大学创建步伐，新创建教育基本现代化县2个。编制实施“健康绍兴”行动计划，深化基层卫生综合改革，推进“双下沉、两提升”长效机制建设，实现国家卫生县（市）全覆盖，建成市立医院、柯桥妇女儿童医院一期等项目。加强公共文化服务体系建设，新建镇街图书分馆15个、文化分馆54个。保护传承优秀地方传统文化，加大绍剧、越剧、莲花落等地方戏曲扶持力度，办好公祭大禹陵、兰亭书法节、越剧巡演等活动。精心举办首届绍兴国际马拉松、皮划艇马拉松世界杯等重大赛事，组织群众性体育活动500场，促进全民健身广泛开展。支持诸暨、嵊州、新昌争创全国县级文明城市。

　　全力打造平安绍兴。坚持发展“枫桥经验”，建设枫桥平安浙江特色小镇，打好平安创建巩固战，打造全省最具安全感城市。加强警务辅助人员队伍建设，深化维稳安保工作机制，着力提升社会治理能力。加强金融风险防控，严厉打击恶意逃废债和非法金融活动。落实初信初访责任制，强化人民调解组织和行业性专业组织功能，基层矛盾调解成功率达到98%。深化“七五”普法，实现四级法律顾问与符合条件法律援助全覆盖。深入开展安全生产“企业主体责任落实年”“行政执法规范建设年”活动，确保事故起数、死亡人数继续“双下降”。实施“创文明交通、治秩序乱象”大会战，打造文明交通“绍兴样本”。加强食品药品安全监管，创建国家食品安全示范城市。

　　为民办实事是市政府长期坚持的重要制度。今年根据省市人大民生实事票决制的要求，市政府通过公开征集，摸排梳理十二方面民生实事候选项目，提请各位代表票决。各级各部门将精心安排、全力实施票决确定的十方面民生实事。

全面加强政府自身建设

　　做好经济社会发展各项工作，必须把政府自身建设放在更加重要的位置，坚持政府过“紧日子”，让群众和企业过“好日子”，努力使政府工作赢得群众信任和支持。

　　勤学习提素质。深入学习贯彻党中央治国理政新理念，切实增强政治意识、大局意识、核心意识、看齐意识。认真贯彻省委省政府和市委决策部署，准确把握政府工作方向和重点。大力推进机关公务员学习制度化、常态化，实施公务员知识更新三年轮训计划，提升干部理论知识素养和解决实际问题能力。大兴调查研究之风，坚持到基层、企业调研学习制度，切实加强新型智库建设。

　　敢担当有作为。牢固树立“实干论英雄、创新论英雄”理念，深入开展“晒亮点、比业绩”，大力弘扬敢于担当、勇于创新、善作善成的精神品质。全面落实改革创新容错免责制度，鼓励创新、宽容失败，真正为担当者担当、为负责者负责，最大限度调动广大干部的积极性、主动性和创造性。坚持和完善工作清单制度，优化督查考核办法，加强追责问责，使政府各项工作有目标、有督查、有问责，确保各项任务落地生根。

　　转作风优服务。扎实开展“勇立潮头创新业、争创一流当标杆”专项行动，把全面从严治党的各项要求落实到政府工作和公务员个人修为的各个环节。持之以恒推动中央“八项规定”精神风化俗成，坚决防止“四风”反弹回潮。切实加强公务员“日志式”工作日程管理，健全考核体系和约束机制。实行公共服务事项标准化管理，完善政务咨询投诉平台功能，深化干部下乡驻村、入企服务和对口帮扶工作，当好企业和群众的“店小二”。

　　强法治促规范。把诚信守法作为法治政府建设的基本要求，着力提升政府公信力。坚持依法行政，自觉接受人大法律监督、政协民主监督，加强政府内部层级监督和审计监督，主动接受社会监督和舆论监督，落实监察体制改革试点任务。务实办理人大代表建议和政协提案，积极参与人大地方立法工作，加大地方法规宣传和施行力度。严格落实行政处罚裁量权基准制度，建立行政复议、行政调解与信访工作衔接机制。健全风险评估、专家咨询、公众参与的重大事项集体决策程序，深入推进政务公开。

　　守底线树形象。把纪律和规矩挺在前面，严格落实各级政府党组主体责任和“第一责任人”责任，着力在常和长、严和实、深和细上下功夫，严守政府工作人员廉洁从政底线，切实做到干净干事。坚持反腐无禁区、全覆盖、零容忍，全面落实“一岗双责”“一案双查”制度，始终保持高压态势，不断深化党风廉政建设和反腐败斗争。加强干部队伍建设，以更严标准打造具有铁一般信仰、铁一般信念、铁一般纪律、铁一般担当的浙江铁军绍兴军团。

　　各位代表，绍兴发展已经站在新的历史起点上。我们要更加紧密地团结在以习近平同志为核心的党中央周围，在省委省政府和中共绍兴市委的坚强领导下，不忘初心、继续前进，以优异的成绩迎接党的十九大和省第十四次党代会胜利召开，为高水平全面建成小康社会、加快建设现代化绍兴而努力奋斗！
